# UBE2J2 is essential for the progression of meiosis prophase I during spermatogenesis in mice

**DOI:** 10.1016/j.isci.2025.112878

**Published:** 2025-06-11

**Authors:** Xiaochen Yu, Jie Cen, Yaxuan Zhang, Tongtong Li, Mingyu Zhang, Fei Gao, Hongbin Liu, Yongzhi Cao

**Affiliations:** 1 Institute of Women, Children and Reproductive Health, Shandong University, Jinan 250012, China; 2 State Key Laboratory of Reproductive Medicine and Offspring Health, Shandong University, Jinan, Shandong 250012, China; 3 Center for Reproductive Medicine, The First Affiliated Hospital, Zhejiang University School of Medicine, Hangzhou 310003, China; 4 National Research Center for Assisted Reproductive Technology and Reproductive Genetics, Shandong University, Jinan, Shandong 250012, China; 5 Key Laboratory of Reproductive Endocrinology (Shandong University), Ministry of Education, Jinan, Shandong 250012, China; 6 Shandong Technology Innovation Center for Reproductive Health, Jinan, Shandong 250012, China; 7 Shandong Provincial Clinical Research Center for Reproductive Health, Jinan, Shandong 250012, China; 8 State Key Laboratory of Stem Cell and Reproductive Biology, Institute of Zoology, Chinese Academy of Sciences, Beijing, China; 9 Model Animal Research Center, Shandong University, Jinan, Shandong 250012, China

**Keywords:** Molecular biology, Cell biology, Organizational aspects of cell biology

## Abstract

The coordination of numerous proteins is necessary for spermatogenesis, including degradation through the ubiquitin-proteasome pathway. Ubiquitin binding enzyme E2 (UBE2J2) is involved in the degradation of endoplasmic reticulum-associated proteins, while its role in spermatogenesis remains unclear. We found that *Ube2j2*^−/−^ mice exhibit azoospermia and spermatocytes undergo meiotic arrest at the mid-pachytene stage. Examining homologous recombination (HR) markers indicated that HR intermediate complexes are unstable and fail to form crossovers in *Ube2j2*^−/−^ spermatocytes. Proteomics analysis uncovered an extensive suite of meiosis- and chromosome segregation–associated proteins unexpressed in mouse spermatocytes lacking functional UBE2J2. Our findings suggest that UBE2J2 could possibly play a key role in SC disassembly, ensuring meiosis can proceed in the late pachynema during male germline cell development in mice, and serves as an essential factor in meiotic recombination and spermatogenesis.

## Highlights

• Ube2j2 knockout results in azoospermia in male mice
• Spermatocytes undergo meiotic arrest at mid-pachynema in *Ube2j2*^−/−^ knockout mice
• *Ube2j2*^−/−^ spermatocytes show obvious proteomic dysregulation

## Graphical abstract

**Figure:**
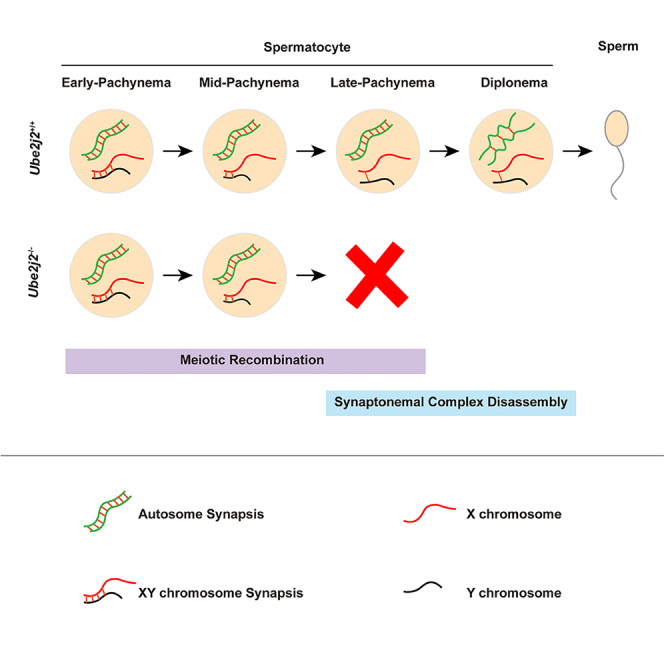

## Introduction

Meiosis is the process of cell division through which diploid germ cells are converted into haploid gametes. The DNA of spermatogonia is replicated once, the cell divides twice in succession, and finally one diploid spermatogonia forms four haploid sperm.^1^ Meiosis is a conserved and specialized form of cell division that separates chromosomes and produces genetically diverse gametes.^2^^,^^3^ The segregation of homologous chromosomes occurs at anaphase in the first meiosis, and sister chromatid separation occurs at anaphase in the second meiosis.^4^ To ensure the proper separation of homologous maternal and paternal chromosomes of origin in the early stages of the first round of meiosis, cells undergo a tightly coordinated series of events, including the formation of double-strand breaks (DSBs) in DNA, homologous chromosome pairing, synapsis, homologous recombination (HR), and crossovers (COs).^5^ The correct and orderly occurrence of these events is required for normal sexual reproduction and, therefore, the study of chromosome behavior during meiosis prophase I is currently a hotspot in genetics research.^6^

Based on morphological changes in chromosomes, meiosis prophase I can be divided into five stages, leptonema, zygonema, pachynema, diplonema, and diakinesis.^7^ Programmed DSBs form in homologous chromosomes at the leptotene stage. After generating programmed DSBs, homologous chromosomes are used as the template for repair to establish a temporary physical connection between homologous chromosomes that promotes the mutual recognition and pairing of homologous chromosomes.^6^ After pairing the homologous chromosomes for synapsis in the leptotene and zygotene stages, the synaptonemal complex (SC) forms between paired chromosomes. After assembly of the full-length SC, spermatocytes enter the pachytene stage.^8^ The HR process is then initiated by the formation of DSBs. DSBs are repaired by excision of the DSBs terminus, homologous chromosome search, DNA single-strand invasion, and double Holliday junctions (dHJs). HR leads to the exchange of homologous DNA fragments, forming COs in the pachytene stage.^9^^,^^10^^,^^11^ Disassembly of the SC must be precisely coordinated with the completion of recombination at the end of pachytene. After COs have formed, the SC is disassembled during diplotene and diakinesis to allow for chromosome segregation at anaphase I.^8^^,^^12^ Defects at any stage in the meiosis prophase I typically lead to impaired synapsis, recombination, segregation of homologous chromosomes, and ultimately, meiotic arrest and sterility.^13^

The orderly progression of meiosis is inseparable from protein synthesis and degradation, which is closely related to the ubiquitin-proteasome system (UPS).^14^^,^^15^ The relationship between the UPS and spermatogenesis has been studied extensively in recent years. Ubiquitination is widespread in the testes,^16^ and defects in the UPS generally result in blocking spermatogenesis, and consequently, male infertility to varying degrees. Among E2 family ligases with a defined role in spermatocyte development, UBE2J1 (ubiquitin-conjugating enzyme E2J1) has been previously found to play a role in male sterility, with male *Ube2j1*^−/−^ mice exhibiting defects in sperm flagellum, acrosome function, and defects in cytoplasmic clearance in elongated sperm cells.^17^ The crucial role of *Ube2j1* in spermatogenesis suggested that other Ube2J family ligases might also participate in male fertility, such as *Ube2j2*. UBE2J2 is a highly conserved E2 protein with a ubiquitin-binding domain and a transmembrane domain that localizes to the endoplasmic reticulum (ER) to participate in ER-associated degradation (ERAD) via recognition and ubiquitination of proteins targeted for proteasomal degradation.^18^^,^^19^ While UBE2J2 has been implicated in human leukocyte antigen (HLA) degradation via binding to the E3 TMEM129 (transmembrane protein 129)^20^^,^^21^; degradation of squalene monooxygenase (SQLE) via binding with the E3, MARCH6 (membrane-associated ring-CH-type finger 6), to maintain homeostasis of cholesterol metabolism homeostasis^22^; regulating Wnt cargo receptor protein, Evi, through binding with the E3, CGRRF1 (cell growth regulator with ring finger domain 1)^23^; and promoting epithelial-stromal transformation and invasion of hepatocellular carcinoma *in vitro*.^24^ However, the potential role of UBE2J2 in male reproduction and sperm development has not been reported.

Based on the data from the transcriptome of various tissues in mice,^25^ we noticed that ubiquitin-conjugating enzyme E2J2 (UBE2J2) expression began to increase significantly in the testis of mice at three days after birth, and this occurred before meiosis initiation (Figure S1A). Therefore, we speculate that UBE2J2 may play a role in mouse spermatogenesis. However, these prior findings represent the full extent of our current knowledge potentially linking UBE2J2 with spermatogenesis, and thus the role of UBE2J2 in spermatogenesis remains entirely unexplored. Here, in the current study, we investigated the role of UBE2J2 in spermatogenesis using gene-edited *Ube2j2*^−/−^ mice. Our results show that male mice lacking UBE2J2 display azoospermia, with meiotic arrest occurring in the mid-pachytene stage of spermatocyte development. In addition, we found that HR intermediates are unstable and appear unable to form COs in *Ube2j2* knockout mouse spermatocytes. In addition, proteomics analysis with immunofluorescent staining suggests that UBE2J2 participates in regulating meiotic HR potentially by promoting SC disassembly. This study thus uncovers a previously unrecognized E2 factor, UBE2J2, involved in meiosis prophase I of spermatogenesis, expanding the scope of our understanding of UBE2J family functions in reproduction.

## Results

### *Ube2j2* knockout results in azoospermia in male mice

**Figure 1:**
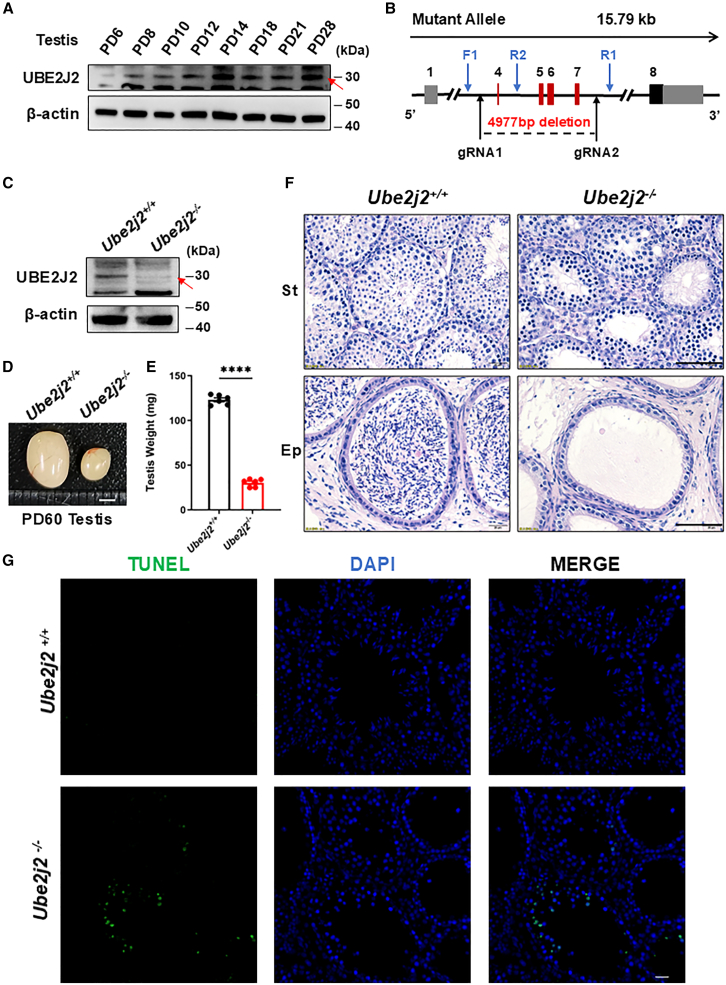
Deletion of UBE2J2 causes male infertility by disrupting spermatogenesis (A) UBE2J2 protein levels in testes of *Ube2j2*^+/+^ mice sampled at the indicated ages. β-actin was used as the loading control. Red arrows indicate the positions of marker bands corresponding to UBE2J2. (B) Schematic representation of the knockout strategy for the *Ube2j2* locus, showing the gRNAs (black arrows), genotyping primers (blue arrows), the coding exons (black and red thick lines), and the non-coding exons (gray thick lines). Red thick lines (coding exons) represent the 4977 bp fragment selectively deleted from the *Ube2j2* locus. (C) Immunoblotting of PD60 *Ube2j2*^−/−^ testes, showing that knockout mice produced no UBE2J2. (D) *Ube2j2*^−/−^ male mice had reduced testis size at PD60 (*n* = 5 *Ube2j2*^+/+^ and *Ube2j2*^−/−^ mice, Welch’s *t*-test: *p* < 0.0001). Scale bar, 2 mm. (E) Quantification of testes weights. Columns display means ± SEM. For both *Ube2j2*^+/+^ and *Ube2j2*^−/−^ group, *n* = 6. ∗∗∗∗, *p* < 0.001, Student’s *t* tests. (F) Hematoxylin staining of testis sections from PD60 *Ube2j2*^−/−^ mice with meiotic arrest in spermatogenesis and empty epididymides. Scale bar, 50 μm. (St) Seminiferous tubules, (Ep) Epididymides. (G) TUNEL assays of apoptotic cells in seminiferous tubules of PD60 *Ube2j2*^−/−^ mice. Scale bar, 20 μm.

To investigate the function of UBE2J2 in reproduction, we detected tissue specificity and expression levels of UBE2J2 throughout early development by immunoblotting in the testes of postnatal male mice (Figure 1A; Figure S1B). Immunoblotting showed that UBE2J2 expression gradually increased in testis samples after the initiation of meiosis on PD8 (postnatal day 8), suggesting a potential meiosis-related function (Figure 1A). Immunoblot assays showed that the large majority of UBE2J2 localized in the cytoplasm in WT, with markedly less, but detectable, nuclear localization (Figure S1C). We then generated *Ube2j2*^−/−^ mice through CRISPR/Cas9-mediated deletion of *Ube2j2* exons 4 to 7 (Figure 1B), and confirmed that UBE2J2 protein was indeed undetectable in the testes of *Ube2j2*^−/−^ mice by immunoblotting (Figure 1C). *Ube2j2*^−/−^ female mice were fertile by conducting a six-month fertility test (Figure S1D). Analysis of gross phenotypes showed no obvious phenotypic alterations except for significantly smaller testes in 2-month-old *Ube2j2*^−/−^ mice compared to those of *Ube2j2*^+/+^ mice (Figures 1D and 1E). In addition, we also assessed mice at postnatal days PD12–14 and found no significant difference in body weight between *Ube2j2*^−/−^ and *Ube2j2*^+/+^ mice (Figure S2A). However, comparison of testis weights and testis weight/body weight ratio showed that both were significantly reduced in PD14 *Ube2j2*^−/−^ mice compared with wild-type (WT) controls, while no such differences were observed in PD12 or PD13 mice (Figures S2B and S2C). Given the known sequence of spermatogenesis events in developing mice,^5^ these findings suggested that loss of UBE2J2 could potentially disrupt spermatogenesis at some point between the leptotene (PD12) and pachytene (PD14) stages.

Based on the above observations, we next compared sperm production between mature *Ube2j2*^−/−^ mice and their WT counterparts. Histological examination of PD60 *Ube2j2*^+/+^ and *Ube2j2*^−/−^ testes and epididymides by hematoxylin staining showed that sperm were completely absent in the epididymis of *Ube2j2*^−/−^ mice, nor were any spermatids present in the seminiferous tubules of these knockout mice (Figure 1F). Further examination of mature male *Ube2j2*^−/−^ mice revealed that spermatogenesis was arrested at the pachytene stage and apoptotic cells could be observed, while no spermatocytes were found in the lumen of some *Ube2j2*^−/−^ seminiferous tubules (Figures 1F and 1G). Analysis of PD13 and PD14 mice showed that spermatocytes were absent in some seminiferous tubules at PD14 in *Ube2j2*^−/−^ testes, whereas no such histological phenotypes were observed in PD13 testes (Figure S2D). These results collectively indicated that UBE2J2 is required for meiosis progression in the male reproductive system of mice.

### *Ube2j2*^−/−^ spermatocytes undergo meiotic arrest at the mid-pachytene stage but exhibit normal DSB formation

**Figure 2:**
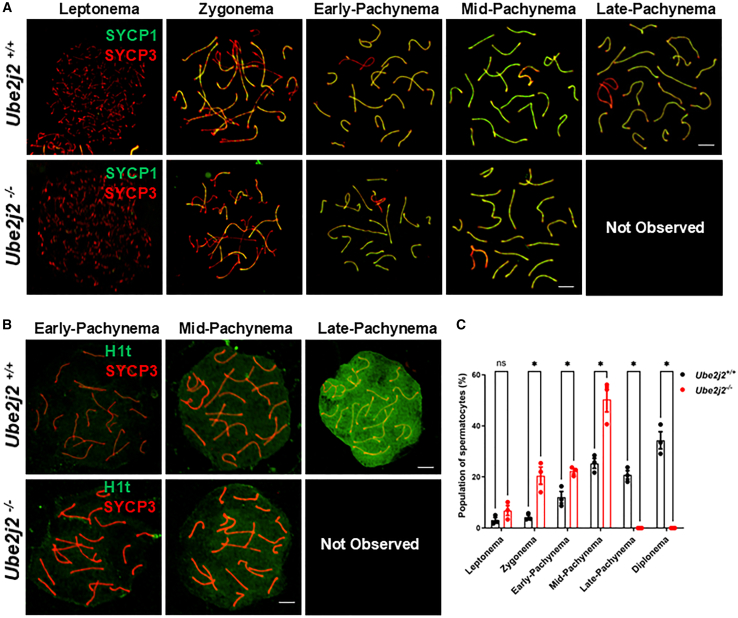
*Ube2j2*^−/−^ mice exhibit meiosis arrest at the mid-pachytene stage (A) Double immunofluorescence labeling of spermatocyte spreads of *Ube2j2*^+/+^ and *Ube2j2*^−/−^ spermatocytes with antibodies against SYCP3 (red) and SYCP1 (green). SYCP3, a marker for the synaptonemal complex lateral element; SYCP1, a marker for the synaptonemal complex central element. Scale bar, 5 μm. (B) Double immunofluorescence labeling of spermatocyte spreads of *Ube2j2*^+/+^ and *Ube2j2*^−/−^ spermatocytes with antibodies against SYCP3 (red) and H1t (green). H1t, a marker for distinguishing the early, mid, and late pachytene stage. H1t signal is absent in the early pachytene stage, but gradually increases beginning in mid-pachynema, reaching highest signal intensity in the late pachytene stage. Scale bar, 5 μm. (C) Quantitative image analysis of cell populations at successive stages of meiotic prophase I in chromosome spreads of spermatocytes. Columns display means ± SEM. Each dot represents an individual mouse. *n* = 3 mice per genotype. Significant differences were determined by two-tailed Student’s *t* test. ∗*p* < 0.05.

We next sought to determine the specific stage in which the absence of UBE2J2 disrupts meiosis progression by staining spermatocyte chromosome spreads from *Ube2j2*^+/+^ and *Ube2j2*^−/−^ mice using established markers for meiotic synapsis and recombination.^13^ We first investigated synapsis in *Ube2j2*^−/−^ mice by co-staining for the SC lateral element marker, SYCP3, and the SC central elements marker, SYCP1. This analysis showed that spermatocytes did not progress to the late pachytene stage in *Ube2j2*^−/−^ mice, although no synapsis abnormalities were observed during any of the stages/steps through which the spermatocytes successfully progressed (Figure 2A). Histone H1t (testis-specific histone H1) is first expressed at mid-pachynema in spermatocyte development, when it replaces the somatic H1 linker histones. Histone H1t then persists into the early stages of elongating spermatids, where it constitutes over half of the total H1 histone complement to condense chromosomes. It is commonly used as a marker for discriminating early from mid-to late-pachytene spermatocytes.^26^ We subsequently found that meiotic progression was arrested at the mid-pachytene stage in *Ube2j2*^−/−^ spermatocytes. Briefly, co-staining for SYCP3 and Histone H1t, to distinguish pachytene sub-stages, showed that no spermatocytes were detectable in the late pachytene or later stages (Figure 2B). We next quantified successive stages of meiotic prophase I in chromosome spreads of spermatocytes and found that the percentages of cells in *Ube2j2*^−/−^ mice at the leptotene, zygotene, early-pachytene and mid-pachytene stages were higher than those in *Ube2j2*^+/+^ mice, whereas *Ube2j2*^−/−^ spermatocytes contained no late-pachytene and diplotene stage spermatocytes (Figure 2C). Thus, the meiotic cell cycle progression appeared retarded and arrested at the mid-pachytene stage in *Ube2j2*^−/−^ spermatocytes. Together, these findings supported the likelihood that murine UBE2J2 performs one or more functions essential for spermatocyte progression through the mid-pachytene stage of meiosis prophase I.

**Figure 3:**
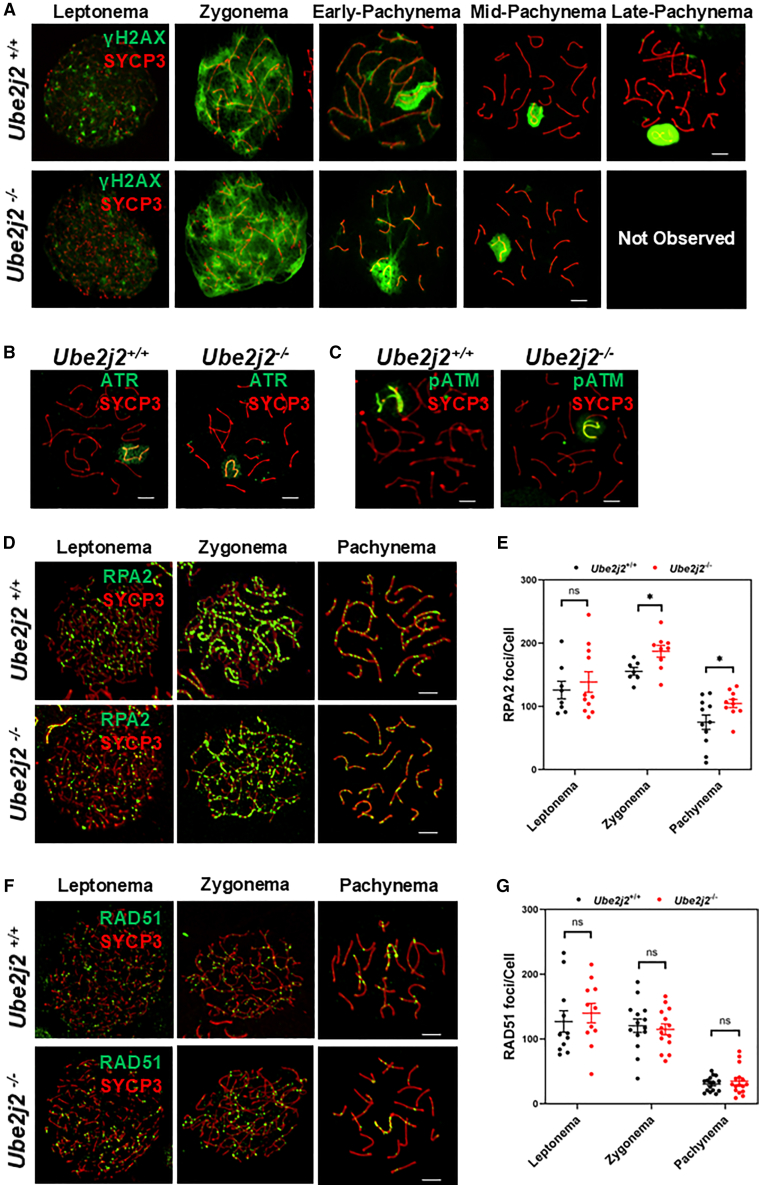
DSBs and DSB repair are normal in *Ube2j2*^−/−^ spermatocytes (A) Double immunofluorescence labeling of spermatocyte spreads of *Ube2j2*^+/+^ and *Ube2j2*^−/−^ spermatocytes with antibodies against SYCP3 (red) and γH2AX (green). γH2AX, a marker for DSB sites. Scale bar, 5 μm. (B) Double immunofluorescence labeling of spermatocyte spreads of *Ube2j2*^+/+^ and *Ube2j2*^−/−^ spermatocytes with antibodies against SYCP3 (red) and ATR (green). ATR, a marker for DNA damage sensing, is required for the second wave of H2AX phosphorylation. Scale bar, 5 μm. (C) Double immunofluorescence labeling of spermatocyte spreads of *Ube2j2*^+/+^ and *Ube2j2*^−/−^ spermatocytes with antibodies against SYCP3 (red) and pATM (green). pATM, a marker for DNA damage sensing, required for first wave of H2AX phosphorylation. Scale bar, 5 μm. (D) Double immunofluorescence labeling of spermatocyte spreads of *Ube2j2*^+/+^ and *Ube2j2*^−/−^ spermatocytes with antibodies against SYCP3 (red) and RPA2 (green). RPA2, ssDNA-binding protein, prevents ssDNA from degrading. Scale bar, 5 μm. (E) The number of RPA2 foci increases from zygotene to pachytene in *Ube2j2*^−/−^ spermatocytes. Columns display means ± SEM. Each dot represents the number of RPA2 foci per nuclei. For *Ube2j2*^+/+^ group, Leptonema: *n* = 8, Zygonema: *n* = 7, Pachynema: *n* = 11. For *Ube2j2*^−/−^ group, Leptonema: *n* = 11, Zygonema: *n* = 9, Pachynema: *n* = 10. ∗, *p* < 0.05, Student’s *t* tests. (F) Double immunofluorescence labeling of spermatocyte spreads of *Ube2j2*^+/+^ and *Ube2j2*^−/−^ spermatocytes with antibodies against SYCP3 (red) and RAD51 (green). RAD51, a marker for ssDNA invasion. Scale bar, 5 μm. (G) *Ube2j2*^−/−^ spermatocytes had comparable numbers of RAD51 foci as compared to *Ube2j2*^+/+^ spermatocytes. Columns display means ± SEM. Each dot represents the number of RAD51 foci per nuclei. For both *Ube2j2*^+/+^ and *Ube2j2*^−/−^ groups, Leptonema: *n* = 11, Zygonema: *n* = 14, Pachynema: *n* = 16. ns, no statistical significance, Student’s *t* tests.

Synapsis and recombination between homologous chromosomes are the main events of meiosis prophase I. As DSB formation is a hallmark of HR in prophase I of meiosis,^27^ we co-stained for SYCP3 and DSB site marker, γH2AX,^11^ which showed no abnormalities in DSB formation in any spermatocyte developmental stages prior to mid-pachytene (Figure 3A). Both ATM and ATR can phosphorylate the serine 139 residue of H2AX protein localized near DSB sites to promote recruitment of DSB repair and HR-related molecules.^28^^,^^29^^,^^30^ Subsequent co-staining against SYCP3 and DSB repair markers, ATR, and pATM (phosphorylated at Ser1981) also indicated that the initiation of DSB repair appeared to occur normally in *Ube2j2*^−/−^ spermatocytes (Figures 3B and 3C).

The first step in DSB repair is resection of DSB ends to produce a 3′ tail on the respective single DNA strands. Single-stranded DNA binding proteins, such as the Replication protein A (RPA) family, RPA2, are recruited to the cleaved single strand to prevent degradation or formation of secondary structures.^31^ Similarly, co-staining for SYCP3 and RPA2 also showed that the location and abundance of RPA2 were normal in the leptotene stage of in *Ube2j2*^−/−^ mice, slightly increasing in the zygotene and pachytene stages (Figures 3D and 3E).

RAD51 binds to ssDNA, replacing RPA, to search for homologous template DNA. DMC1 then interacts with RAD51 to invade homologous chromosomes through DMC1-ssDNA nucleoprotein filament, forming a nascent joint-molecule (JM) intermediate called a D loop.^32^^,^^33^^,^^34^^,^^35^ Moreover, co-staining for SYCP3 and RAD51, a marker of single strand invasion, showed that the number of RAD51 foci on chromosomes in *Ube2j2*^−/−^ spermatocytes did not obviously differ from that of controls (Figures 3F and 3G). These results suggested that UBE2J2 could potentially affect the single-strand protection process, but not the single-strand invasion process.

### *Ube2j2* knockout spermatocytes have unstable recombination intermediates and fail to form crossovers

**Figure 4:**
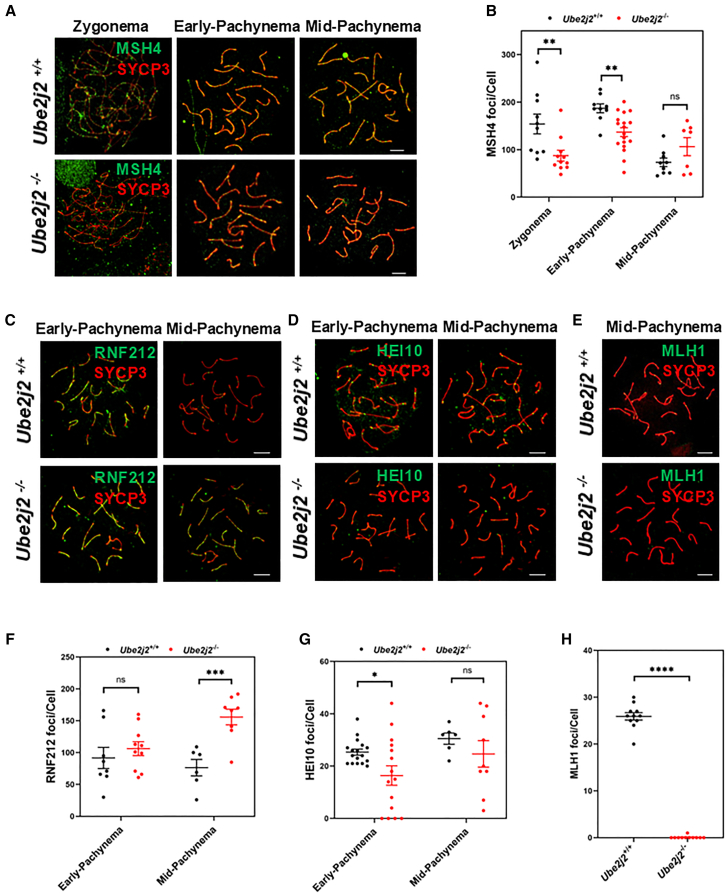
*Ube2j2*^−/−^ spermatocytes display unstable recombination intermediates and fail to form crossovers (A) Double immunofluorescence labeling of spermatocyte spreads of *Ube2j2*^+/+^ and *Ube2j2*^−/−^ spermatocytes with antibodies against SYCP3 (red) and MSH4 (green). MSH4, a marker for stabilizing recombination intermediates to promote crossover formation. Scale bar, 5 μm. (B) The number of MSH4 foci decreases from zygotene to early-pachytene in *Ube2j2*^−/−^ spermatocytes. Columns display means ± SEM. Each dot represents the number of MSH4 foci per nuclei. For *Ube2j2*^+/+^ group, Zygonema: *n* = 10, early-Pachynema: *n* = 9, mid-Pachynema: *n* = 9. For *Ube2j2*^−/−^ group, Zygonema: *n* = 11, early-Pachynema: *n* = 18, mid-Pachynema: *n* = 7. ∗∗, *p* < 0.01. ∗∗∗∗, *p* < 0.001, Student’s *t* tests. (C) Double immunofluorescence labeling of spermatocyte spreads of *Ube2j2*^+/+^ and *Ube2j2*^−/−^ spermatocytes with antibodies against SYCP3 (red) and RNF212 (green). RNF212, a marker for crossover-fated intermediates stabilization. Scale bar, 5 μm. (D) Double immunofluorescence labeling of spermatocyte spreads of *Ube2j2*^+/+^ and *Ube2j2*^−/−^ spermatocytes with antibodies against SYCP3 (red) and HEI10 (green). HEI10, a marker for recombination intermediates stabilization, promoting RNF212 and/or MSH4-MSH5 turnover from chromosomes. Scale bar, 5 μm. (E) Double immunofluorescence labeling of spermatocyte spreads of *Ube2j2*^+/+^ and *Ube2j2*^−/−^ spermatocytes with antibodies against SYCP3 (red) and MLH1 (green). MLH1, a marker for crossovers. Scale bar, 5 μm. (F) The number of RNF212 foci increases at mid-pachytene in *Ube2j2*^−/−^ spermatocytes. Columns display means ± SEM. Each dot represents the number of RNF212 foci per nuclei. For *Ube2j2*^+/+^ group, early-Pachynema: *n* = 8, mid-Pachynema: *n* = 6. For *Ube2j2*^−/−^ group, early-Pachynema: *n* = 10, mid-Pachynema: *n* = 8. ∗∗∗, *p* < 0.005, Student’s *t* tests. (G) The number of HEI10 foci decreases at early-pachytene in *Ube2j2*^−/−^ spermatocytes. Columns display means ± SEM. Each dot represents the number of HEI10 foci per nuclei. For *Ube2j2*^+/+^ group, early-Pachynema: *n* = 17, mid-Pachynema: *n* = 6. For *Ube2j2*^−/−^ group, early-Pachynema: *n* = 15, mid-Pachynema: *n* = 9. ∗, *p* < 0.05, Student’s *t* tests. (H) The number of MLH1 foci decreases in *Ube2j2*^−/−^ spermatocytes. Columns display means ± SEM. Each dot represents the number of MLH1 foci per nuclei. For both *Ube2j2*^+/+^ and *Ube2j2*^−/−^ group, *n* = 11. ∗∗∗∗, *p* < 0.001, Student’s *t* tests.

Given the above results showing that UBE2J2 does not affect DSB formation but is required for progression through the mid-pachytene stage, we next investigated whether subsequent HR processes were affected by *Ube2j2* knockout. To this end, we examined three proteins, including MSH4, RNF212, and HEI10, that are known to be recruited to HR repair sites on the recombination intermediates in preparation for subsequent COs.^13^^,^^36^^,^^37^^,^^38^ Quantitative analysis of immunostaining imaging data showed that *Ube2j2*^−/−^ spermatocytes had significantly fewer MSH4 foci from the zygotene to early-pachytene stages compared to that in *Ube2j2*^*+/+*^ spermatocytes (Figures 4A and 4B). As Msh4 and Msh5 constitute the MutSγ complex, which stabilizes recombination intermediates and facilitates their resolution into COs,^35^ these results suggested that recombination intermediates might exhibit decreased stability in *Ube2j2*^−/−^ spermatocytes. Moreover, we also detected a significant decrease in the number of HEI10 foci, which promotes COs at sites that are predominantly stabilized by RNF212,^39^ on *Ube2j2*^−/−^ spermatocytes at the early-pachytene stage, while RNF212 accumulation was significantly increased in the mid-pachytene *Ube2j2*^−/−^ spermatocytes compared to corresponding stage *Ube2j2*^+/+^ spermatocytes (Figures 4C, 4D, 4F, and 4G). Finally, these staining assays also showed that RNF212 failed to dissociate normally from *Ube2j2*^−/−^ chromosomes and that HEI10 was absent at the pachytene stage, suggesting impaired stability of recombination intermediates, resulting in the absence of crossover marker, MLH1,^13^ on chromosomes of *Ube2j2*^−/−^ mice (Figures 4E and 4H). These results suggested that HR was impaired in *Ube2j2*^−/−^ spermatocytes, with intermediates failing to form COs and complete recombination.

### Differential expression of homologous chromosome-segregation-related proteins in *Ube2j2*^−/−^ testes

Since UBE2J2 is a ubiquitin-binding enzyme, we evaluated the overall ubiquitination levels in spermatocytes in various stages of meiosis prophase I. Immunofluorescence co-staining for SYCP3 and ubiquitin marker, FK2, showed no apparent differences between *Ube2j2*^+/+^ and *Ube2j2*^−/−^ mice, which was further verified by western blot detection with an anti-ubiquitin antibody in testes extracts (Figures S2E and S2F). Recalling the difference in phenotypes between PD13 and PD14 *Ube2j2*^−/−^ testes (Figures S2A–S2D), we subsequently conducted proteomics analysis by mass spectrometry to identify differentially accumulating or depleted proteins in PD13 *Ube2j2*^−/−^ testes that might contribute to the meiotic disruption observed in PD14 testes.

**Figure 5:**
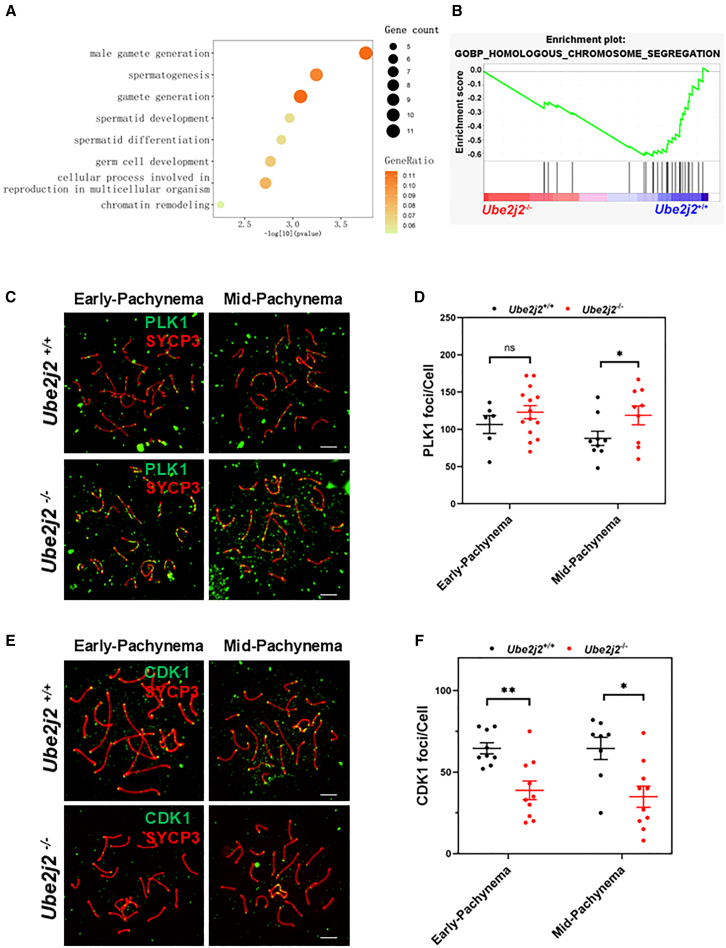
Abnormal nuclear recruitment of the synaptonemal complex disassembly molecules in *Ube2j2*^−/−^ spermatocytes (A) GO enrichment analysis of differentially expressed downregulated proteins in *Ube2j2*^−/−^ testes. (B) Gene set enrichment analysis (GSEA) analysis of differentially expressed proteins. (C) Double immunofluorescence labeling of spermatocyte spreads of *Ube2j2*^+/+^ and *Ube2j2*^−/−^ spermatocytes with antibodies against SYCP3 (red) and PLK1 (green). Scale bar, 5 μm. (D) The number of PLK1 foci increases at mid-pachytene in *Ube2j2*^−/−^ spermatocytes. Columns display means ± SEM. Each dot represents the number of PLK1 foci per nuclei. For *Ube2j2*^+/+^ group, early-Pachynema: *n* = 6, mid-Pachynema: *n* = 9. For *Ube2j2*^−/−^ group, early-Pachynema: *n* = 14, mid-Pachynema: *n* = 9. ∗, *p* < 0.05, Student’s *t* tests. (E) Double immunofluorescence labeling of spermatocyte spreads of *Ube2j2*^+/+^ and *Ube2j2*^−/−^ spermatocytes with antibodies against SYCP3 (red) and CDK1 (green). Scale bar, 5 μm. (F) The number of CDK1 foci decreases from early-pachytene to mid-pachytene in *Ube2j2*^−/−^ spermatocytes. Columns display means ± SEM. Each dot represents the number of CDK1 foci per nuclei. For *Ube2j2*^+/+^ group, early-Pachynema: *n* = 9, mid-Pachynema: *n* = 8. For *Ube2j2*^−/−^ group, early-Pachynema: *n* = 10, mid-Pachynema: *n* = 10. ∗, *p* < 0.05. ∗∗, *p* < 0.01, Student’s *t* tests.

First, analysis of differentially down-regulated proteins in proteomics data from PD13 *Ube2j2*^+/+^ and *Ube2j2*^−/−^ testes revealed enrichment in GO terms such as “male gamete generation,” “spermatogenesis,” “germ cell development,” and “chromatin remodeling,” among others (Figure 5A). Further analysis identified 168 proteins, from among the 5,791 total identified proteins, that were exclusively expressed in *Ube2j2*^+/+^ testes, and 53 proteins uniquely expressed in testes of *Ube2j2*^−/−^ mice (Figure S3A). The subset of proteins exclusively associated with WT testes was enriched in GO terms including “ubiquitination modification,” “spermatogenesis,” and “chromatin structure” (Figure S3B), while proteins enriched in the *Ube2j2*^−/−^ testes had annotations related to “steroid anabolic pathway” (Figure S3C).

Notably, measurement of serum steroids in PD13 mice revealed that corticosterone and cortexolone levels were significantly higher in *Ube2j2*^−/−^ mice compared to their WT counterparts. In contrast, androgen and estrogen levels did not significantly differ (Figure S4). Subsequent gene set enrichment analysis (GSEA) indicated that proteins associated with the separation of homologous chromosomes were predominantly enriched in the *Ube2j2*^+/+^ group, and were generally downregulated in the *Ube2j2*^−/−^ group compared to wild-type *Ube2j2*^+/+^ controls (Figure 5B).

These exploratory analyses thus uncovered decreases in proteins associated with spermatogenesis and chromatin remodeling processes in PD13 *Ube2j2*^−/−^ mice testes compared to their WT littermates at the same timepoint. Additionally, several proteins involved in segregation of homologous chromosomes were downregulated in *Ube2j2*^−/−^ testes. Viewed alongside our findings of spermatogenic arrest at the mid-pachytene stage, these results indicated that UBE2J2 is required for homologous chromosome segregation in spermatocyte development in mice.

### UBE2J2 may participate in promoting synaptonemal complex disassembly to regulate meiotic recombination

Given the above findings, we then explored the possible role of UBE2J2 in homologous chromosome segregation. As both PLK1 (Polo-like kinase 1) and the CDK1-Cyclin B complex are required for SC depolymerization in the late pachytene stage,^40^^,^^41^^,^^42^ we next examined PLK1 and CDK1 by immunofluorescent staining in *Ube2j2*^−/−^ and WT mice. Subcellular localization assays of PLK1 and CDK1 indicated that the number of PLK1 foci on SCs significantly increased in *Ube2j2*^−/−^ spermatocytes compared to *Ube2j2*^+/+^ spermatocytes (Figures 5C and 5D), whereas the number of CDK1 foci significantly decreased (Figures 5E and 5F), suggesting that SC disassembly was possibly defective in *Ube2j2*^−/−^ spermatocytes. Overall, these results indicated that SC disassembly is blocked in *Ube2j2*^−/−^ mouse spermatocytes, and imply that UBE2J2 may be required for SC disassembly to ensure meiosis can proceed in the late pachytene stage in the development of male germline cells in mice.

## Discussion

In this study, we found that UBE2J2 plays a vital role in the first meiotic cell division in spermatocytes of mice by regulating HR of chromosomes, potentially by contributing to disassembly of the SC. We found that deletion of *Ube2j2* results in azoospermia in male mice and spermatocyte meiosis is arrested in the mid-pachytene stage. *Ube2j2* knockout does not affect the formation of DSBs or repair initiation, nor does it affect the synapsis of homologous chromosomes, but it can lead to unstable intermediates in HR and failure to form COs. Functional enrichment analysis of proteomics data indicated that proteins involved in spermatogenesis, cell cycle, and chromosome segregation pathways are all downregulated in UBE2J2 knockout spermatocytes, while steroid anabolic pathway was enriched but the synthesis of sex hormones was not affected. We found that UBE2J2 could potentially play a role in SC disassembly and HR.

Synapsis and recombination are key events in meiosis prophase I. Immunofluorescent staining for HR and synapsis markers showed that *Ube2j2*^−/−^ spermatocytes exhibit normal synapsis, DSB formation, initiation of DSB repair, and single strand invasion processes. However, we did note an increasing trend in the single strand protection marker, RPA2, suggesting possible dysregulation. However, no aberrant phenotype was associated with single-strand invasion, possibly due to compensation for this potential dysregulation. Notably, increased levels of RNF212 and its failure to dissociate from *Ube2j2*^−/−^ chromosomes, combined with diminished levels of HEI10, together suggested that intermediates of HR were unstable in the absence of UBE2J2. Additionally, the CO marker, MLH1, was absent from chromosomes in *Ube2j2*^−/−^spermatocytes, suggesting that CO was also abolished by UBE2J2 knockout. MSH4 and MSH5 constitute the MutSγ complex, which is known to stabilize HR intermediates and promote its disintegration into COs.

As synapsis ensues, binding to the MutSγ complex initially stabilizes most or all D loops.^39^ At crossover sites, RNF212-dependent SUMOylation enhances association with MutSγ, stabilizing D loops, and thus allowing formation of crossover-specific dHJ.^39^ Colocalization between MSH4 and RNF212 is required for RNF212-dependent SUMOylation of MSH4, which protects MSH4 from degradation,^13^ while HEI10 and RNF212 are mutually antagonistic.^43^ HEI10 and RNF212 have been shown to co-localize at the recombination site where COs form in the mid-pachytene stage,^39^^,^^43^ and HEI10 inhibits the stabilizing effect of RNF212 on MutSγ molecules. This function thus accelerates the dissociation of RNF212 and MutSγ proteins from the SC, promoting the turnover of recombination-associated proteins.^43^^,^^44^ Our results show that MSH4 foci is decreased in *Ube2j2*^−/−^ spermatocytes and suggest that HR intermediates are unstable. Even if reduced HEI10 levels and increased RNF212 together can stabilize the MutSγ complex, the localization of MSH4 to the SC cannot be restored, while destabilization of HR intermediates could block the occurrence of COs.

It also warrants noting that HEI10 and RNF212 localization can indicate a likely CO site. In *Ube2j2*^−/−^ spermatocytes, HEI10 was greatly reduced in the early-pachytene phase, while RNF212 was widely distributed throughout the SC and did not dissociate from the chromosomes in time for CO formation at the mid-pachytene stage. This functional dysregulation likely destabilized HR intermediates in *Ube2j2*^−/−^ spermatocytes, in turn preventing CO formation. These results imply the possible disruption of HR intermediate-related protein expression in *Ube2j2*^−/−^spermatocytes, and suggest that UBE2J2 plays a role in stabilizing HR intermediates.

Although UBE2J2 is a ubiquitin-binding enzyme, the overall ubiquitination levels in spermatocytes and testes did not significantly change in *Ube2j2*^−/−^ mice compared with WT. We therefore conducted proteomics analysis of testes from *Ube2j2*^+/+^ and *Ube2j2*^−/−^ mice to screen for proteins and pathways that might contribute to the observed changes in specific proteins. Functional annotation and enrichment analysis indicated that *Ube2j2* knockout was associated with abnormal segregation of homologous chromosomes. Homologous chromosome segregation and COs exert mutually positive effects in which COs are produced by dissociation of HR intermediates, and SC depolymerization occurs after CO formation. Initiation of SC depolymerization requires phosphorylation of proteins in the central and lateral SC axis by PLK1 and the CDK1-Cyclin B complexes.^8^^,^^40^ Previous studies have shown that mutants that affect CDK1 activation can cause abnormal SC disassembly and subsequent meiotic arrest in the mid-late pachytene stages.^41^^,^^42^^,^^45^^,^^46^^,^^47^ CDK1 must be complexed with cyclin B1 to be active.^48^ Before CDK1 can interact with Cyclin B1, CDK1 should interact with the chaperone protein HSPA2 (formerly HSP70-2) to get activated.^45^ HSPA2 localizes to the SC, and it is possible that CDK1 is recruited to the SC through its interaction with HSPA2.^12^ Previous studies have detected CDK1 in the centromeres of metaphase I chromosome in mouse spermatocytes.^48^ In this study, we found scattered SC localization of CDK1 in mice pachytene stage spermatocytes. Our results show that PLK1 foci counts were increased in the SC of *Ube2j2*^−/−^ spermatocytes, while CDK1 foci counts were significantly decreased. Western blots also indicated that UBE2J2 was expressed in the nucleus of WT, but not *Ube2j2*^−/−^ spermatocytes, suggesting that UBE2J2 might promote SC disassembly. The increased expression of PLK1 in the SC of *Ube2j2*^−/−^ spermatocytes may thus reflect compensation for the failure of CDK1-mediated SC disassembly.

The subset of proteins exclusively associated with *Ube2j2*^−/−^ testes was enriched in GO terms related to “steroid anabolic pathway”. Thus, we measured the serum steroids in PD13 mice and revealed that corticosterone and cortexolone levels were significantly higher in *Ube2j2*^−/−^ mice compared to their WT counterparts. In contrast, androgen and estrogen levels did not significantly differ (Figure S4). The pathway of steroid hormone production is commonly divided into the synthesis of mineralocorticoid, glucocorticoids and sex steroids.^49^ Corticosterone is the intermediate product in aldosterone synthesis pathway, which belongs to mineralocorticoid and regulates sodium balance and blood pressure.^49^^,^^50^^,^^51^ Cortexolone, also known as 11-deoxycortisol, is the immediate precursor of cortisol, which are glucocorticoids involving in a wide array of metabolic, anti-inflammatory, immunosuppressive, and cognitive signaling processes.^49^^,^^52^^,^^53^ Since the sex steroids pathway of *Ube2j2*^−/−^ mice was not affected, the changes of mineralocorticoid and glucocorticoids did not directly affect sex steroids and spermatogenesis, so the changes of corticosterone and cortexolone were not further studied in this study.

The global infertility rate among reproductive age couples can reach 15%, nearly 50% of which is due to male factors.^54^^,^^55^ Despite advances in our understanding of genetic factors related to abnormal spermatogenesis, there remain numerous genes associated with azoospermia that have yet to be discovered. In this study, we explored the role of UBE2J2 in spermatogenesis and found that UBE2J2 is essential for HR, potentially by stabilizing HR intermediates, and possibly promoting SC disassembly in mouse spermatocytes. It is highly plausible that a human family could carry mutation in *Ube2j2* gene that manifests as azoospermia. Our current study can provide valuable guidance for developing targeted therapeutic options for such cases.

### Limitations of the study

One shortcoming of this study is that no protein substrate of UBE2J2 ubiquitination modification in the traditional ERAD pathway was identified in spermatocyte meiosis. Since ERAD activities occur in the cytoplasm, and currently there is no published evidence indicating a direct relationship between ERAD and meiosis, it is challenging to link ERAD to chromosomal behavior within the nucleus. Whether UBE2J2 affects the meiosis process through ubiquitination of regulatory targets in spermatocytes requires further investigation. Another shortcoming of this study is that the specific mechanism or direct function of UBE2J2 in spermatocytes remains unclear. Our proteomics data, together with immunofluorescent staining assays for the known SC disassembly proteins, PLK1 and CDK1, suggest that UBE2J2 could potentially play a role in SC disassembly. We observed that *Ube2j2*^−/−^ spermatocyte populations show complete arrest at mid-pachynema, with no spermatocytes detectable in late pachynema or subsequent stages. Although SC disassembly does not begin at the mid-pachytene stage, some proteins required for this process may be already expressed in spermatocytes in preparation for this event, which occurs in the next stage. However, our immunoprecipitation-mass spectrometry (IP-MS) and co-immunoprecipitation assays aimed at identifying substrates of UBE2J2 were thus far unsuccessful in identifying such interaction partners, precluding our ability definitively determine the mechanism through which UBE2J2 knockout results in disrupting meiosis in spermatogenesis.

## Resource availability

### Lead contact

Requests for further information and resources should be directed to and will be fulfilled by the lead contact, Yongzhi Cao (yzcao@sdu.edu.cn).

### Materials availability

Mouse lines generated in this study will be made available on request, but we may require a payment and/or a completed materials transfer agreement if there is potential for commercial application.

### Data and code availability

Proteomic data have been deposited at ProteomeXchange and are publicly available as of the date of publication.

The accession number for the raw and processed data files is PXD063637.

Any additional information required to reanalyze the data reported in this paper is available from the lead contact upon request.

## Acknowledgments

We appreciate the support of the Translational Medicine Core Facility of Shandong University for consultation and instrument use.

This work was supported by the Major Innovation Projects in Shandong Province [2021ZDSYS16], and Science Foundation for Distinguished Young Scholars of Shandong [ZR2021JQ27], and Taishan Scholars Program for Young Experts of Shandong Province [tsqn202103192], and 10.13039/501100012166National Key Research and Development Program of China [2023YFC2706405].

## Author contributions

Conceptualization, Y.-Z.C., H.-B.L., and F.G.; data curation, X.-C.Y. and J.C.; funding acquisition, Y.-Z.C., H.-B.L., and F.G.; investigation, X.-C.Y. and J.C.; methodology, X.-C.Y., J.C., Y.-X.Z., T.-T.L., and M.-Y.Z.; validation, X.-C.Y. and J.C.; Visualization, X.-C.Y., J.C., and T.-T.L.; project administration, Y.-Z.C. and H.-B.L.; supervision, Y.-Z.C., H.-B.L., and F.G.; writing – original draft, X.-C.Y. and J.C.; writing—review and editing, X.-C.Y., J.C., and Y.-Z.C. All authors have read and agreed to the published version of the manuscript.

## Declaration of interests

The authors declare no conflict of interest.

## STAR★Methods

### Key resources table

**Table:** 

REAGENT or RESOURCE	SOURCE	IDENTIFIER
**Antibodies**		

anti-UBE2J2 polyclonal antibody	This paper; generated by Dia-an Biological Technology Incorporation	Cat#C1325
Beta Actin antibody	Proteintech	Cat#66009-1-Ig; RRID: AB_2687938
Lamin B1 antibody	Proteintech	Cat#12987-1-AP; RRID: AB_2136290
Ubiquitin (P4D1) Mouse mAb	Cell Signaling Technology	Cat#3936; RRID: AB_331292
SCP3 antibody	Abcam	Cat#ab15093; RRID: AB_301639
SCP1 antibody	Abcam	Cat#ab15090; RRID: AB_301636
HIST1H1T antibody	Abcam	Cat#ab81498; RRID: AB_1860528
Anti-phospho-Histone H2A.X (Ser139) Antibody, clone JBW301	Millipore	Cat#05-636; RRID: AB_309864
Anti-ATR Antibody (C-1)	Santa Cruz Biotechnology	Cat#sc-515173; RRID: AB_2893291
Anti-phospho-ATM (Ser1981), clone 10H11.E12	Millipore	Cat#05-740; RRID: AB_309954
RPA32/RPA2 antibody [EPR2877Y]	Abcam	Cat#ab76420; RRID: AB_1524336
RAD51 Polyclonal Antibody	Thermo Fisher Scientific	Cat#PA5-27195; RRID: AB_2544671
MSH4 antibody	Abcam	Cat#ab58666; RRID: AB_881394
RNF212 antibody	gifted from Dr. Mengcheng Luo, Wuhan University	N/A
CCNB1IP1 antibody	Abcam	Cat#ab71977; RRID: AB_1310045
Mouse Anti-MLH-1 Monoclonal Antibody, Unconjugated, Clone G168-15	BD Biosciences	Cat#550838; RRID: AB_2297859
Mono and Polyubiquitylated Conjugates mAb (clone FK2)	Ubiquigent	Cat#68-0121-500
CDK1 antibody [A17]	Abcam	Cat#ab18; RRID: AB_2074906
PLK1 antibody [35-206]	Abcam	Cat#ab17056; RRID: AB_443612
Goat Anti-Rabbit IgG H&L (Alexa Fluor® 488)	Abcam	Cat#ab150077; RRID: AB_2630356
Goat Anti-Mouse IgG H&L (Alexa Fluor® 594) preadsorbed	Abcam	Cat#ab150120; RRID: AB_2631447
Goat Anti-Rabbit IgG H&L (Alexa Fluor® 594)	Abcam	Cat#ab150080; RRID: AB_2650602
Goat Anti-Mouse IgG H&L (Alexa Fluor 488) preadsorbed Antibody	Abcam	Cat#ab150117; RRID: AB_2688012

**Chemicals, peptides, and recombinant proteins**		

RIPA buffer	Beyotime Biotechnology	Cat# P0013B
PMSF	Beyotime Biotechnology	Cat#ST506
5×SDS-PAGE protein loading buffer	Beyotime Biotechnology	Cat# P0015L
Bouin’s solution	Sigma-Aldrich	Cat#HT10132
Paraformaldehyde,4%	Solarbio	Cat#P1110
Triton X-100	Sigma-Aldrich	Cat#T9284
DAPI	Abcam	Cat#ab104139
Protease Inhibitor Cocktail III	Merck Millipore	Cat#539134
trichloroacetic acid	Sigma-Aldrich	Cat#T4885
Triethylammonium bicarbonate buffer	Sigma-Aldrich	Cat#T7408

**Critical commercial assays**		

BCA Protein Assay Kit	Beyotime Biotechnology	Cat#P0011

**Deposited data**		

Proteomic data of testis from WT and *Ube2j2*^−/−^ mice	ProteomeXchange	PXD063637

**Experimental models: Organisms/strains**		

Mouse: C57BL/6JNifdc	Beijing Vital River Laboratory Animal Technology Co.	Strain Code: 219
Mouse: rl:CD1(ICR)	Beijing Vital River Laboratory Animal Technology Co.	Strain Code: 201

**Oligonucleotides**		

gRNA targeting sequence for Ube2j2 knockout: gRNA1: ATCCTTCTCTCCCGGGAGTTTGG; gRNA2: AGCTGTCTTTCCCGCACTATAGG	This paper	N/A
Primer for the Ube2j2 mutant and wild-type allele: Forward (F): 5′- GTTTATGCACCTGTCTCCTCCAG-3′	This paper	N/A
Primer for the Ube2j2 mutant allele: Reverse 1 (R1): 5′-CCATTTGGGAGCAGAACTCAAG-3′	This paper	N/A
Primer for the Ube2j2 wild-type allele: Reverse 2 (R2): 5′-TCTAGAACTCCAGATACTGACCC-3′	This paper	N/A

**Software and algorithms**		

GraphPad Prism	GraphPad	RRID: SCR_002798
Bitplane Imaris	Imaris	RRID:SCR_007370

### Experimental model and study participant details

The *Ube2j2* gene (NCBI Reference Sequence: NM_021402; Ensembl: ENSMUSG00000023286) is located on chromosome 4 in mice. *Ube2j2* contains eight exons, with the ATG start codon located in exon 2 and the TGA stop codon located in exon 8 (Transcript: ENSMUST00000024056). The *Ube2j2*-deficient mice were generated in the C57BL/6 genetic background by deleting a genomic DNA fragment spanning exons 4 to 7 through CRISPR/Cas9-mediated genome editing (Cyagen Biosciences). The gRNA target sequences used for *Ube2j2* knockout included gRNA1 (ATCCTTCTCTCCCGGGAGTTTGG, matching the complementary strand) and gRNA2 (AGCTGTCTTTCCCGCACTATAGG, also matching the complementary strand). The founder mice were genotyped by PCR and Sanger DNA sequencing using genomic DNA extracted from mouse tails. The PCR primers used to amplify the *Ube2j2* mutant allele were Forward 1 (F1): 5′- GTTTATGCACCTGTCTCCTCCAG-3′ and Reverse 1 (R1): 5′-CCATTTGGGAGCAGAACTCAAG-3′, yielding a 556 bp fragment. Amplification of the *Ube2j2* wild-type allele was performed using the above F1 primer with Reverse 2 (R2): 5′-TCTAGAACTCCAGATACTGACCC-3′, yielding a 699 bp fragment. To observe more pronounced fertility phenotypes under experimental conditions, gene edited *Ube2j2*^−/−^ female mice were crossed with male wild-type (WT) ICR. Heterozygous offspring were then crossed for up to 10 generations to obtain *Ube2j2*^−/−^ homozygous mice for experimentation. Wild-type C57BL/6 and ICR mice for propagation were purchased from Charles River Laboratories, Beijing, China. All mice were housed under controlled environmental conditions with free access to water and food, and illumination from 6 a.m. to 6 p.m. All experimental protocols were approved by the Animal Ethics Committee of the School of Medicine, Shandong University (ethical committee reference number 2021 #95).

### Method details

#### Antibodies

The rabbit anti-UBE2J2 polyclonal antibody used for Western blotting (1:1,000 dilution) was generated by Dia-an Biological Technology Incorporation (Wuhan, China); full-length recombinant mouse UBE2J2 protein was used as the source of antigens, with the following peptide sequence: ERTVILPQKPLLCVPSASCDLPSPVTLDELSALTPV NSICSVQGTVVDVDESTAFSWPVCDRCGNGRLEQKPEDGGTFSCGDCSQLVLSPLQERHLHVFLDCPTRPESTVKVKLLESSISLLLMSAASEDGSYEVESVLGKEMGPLLCFVQSITTQQSSCVVTL.

#### Immunoblotting

To prepare protein extracts, testis extracts were prepared using a homogenizer in RIPA buffer (Beyotime Biotechnology, P0013B) supplemented with 100 mM PMSF (Beyotime Biotechnology, ST506). After homogenization, the testes lysates were incubated on ice for 30 min and then centrifuged at 4°C, 12,000 rpm for 20 min. The supernatant was transferred to a new tube and 5 × SDS-PAGE protein loading buffer (Beyotime Biotechnology, P0015L) was added. After boiling at 95°C for 10 min, the protein lysates were used for Western blots.

#### Fertility tests

To test for alterations in female fertility, five-week-old female mice (n = 3) of *Ube2j2*^+/+^ and *Ube2j2*^−/−^ genotype were caged with a single wild-type C57BL/6 male with normal fertility for 6 months. The number of pups were recorded for each pregnancy and the cumulative pup numbers produced by each female compared at the end of the study period.

#### Tissue collection and histological analysis

Testes and epididymides from more than three mice for each genotype were dissected immediately after euthanasia, fixed in Bouin’s solution (Sigma-Aldrich, HT10132) overnight, stored in 70% ethanol, and embedded in paraffin after dehydration. The 5 μm sections were prepared and mounted on glass slides. After deparaffinization, slides were stained with hematoxylin for histological analysis. Histology was conducted with an epifluorescence microscope (BX52, Olympus).

#### Spermatocyte chromosome spreads

Testes were dissected and seminiferous tubules were incubated in hypotonic extraction buffer (30 mM Tris, 50 mM sucrose, 17 mM trisodium citrate, 5 mM ethylenediaminetetraacetic acid, 0.5 mM dithiothreitol; adjusted to pH 8.2) for 30 to 40 min at room temperature. Subsequently, the tubules were cut into pieces in 100 mM sucrose at pH 8.2. The cell suspensions were then loaded on slides containing fixation solution (1% paraformaldehyde and 0.15% Triton X-100, pH 9.2) and dried in a humid chamber at room temperature for 4 to 5 h prior to storage −80°C.

#### Immunofluorescence staining assays

The slides were transferred to PBST solution containing 0.1% Triton X-100 and incubated for 10 to 15 min at room temperature. After washing in PBS, the slides were blocked in 5% BSA solution for 30 min at room temperature. The primary antibodies were incubated overnight at 4°C, then washed again in PBS. Prior to observation, the slides were incubated in Alexa-conjugated secondary antibodies at room temperature for 30 min, then mounted with coverslips using a mounting medium with DAPI (Abcam, ab104139). Immunolabeled chromosome spreads were imaged by confocal microscopy using a Leica TCS SP5 resonant-scanning confocal microscope driven by Leica software. The Andor Dragonfly spinning disc confocal microscope was driven by Fusion Software, and projection images were prepared using Bitplane Imaris (version 8.1) software.

#### Serum steroid hormone measurement

Blood samples were collected by retro-orbital bleeding and left at room temperature for 30 min, then incubated at 4°C for 1 h. Samples were centrifuged at 3000 rpm for 20 min at 4°C. The supernatant was transferred to a new microcentrifuge tube and centrifuged at 3000 rpm for 5 min at 4°C. The supernatant was then transferred to a new tube and stored at −80°C. After the six *Ube2j2*^+/+^ and six *Ube2j2*^−/−^ mice samples were collected, serum samples were packed on dry ice and sent to Bomiao Biological Company for measurement of steroid hormones.

#### Proteomics analysis

For proteomics analysis, testes of *Ube2j2*^−/−^ and WT mice were collected on ice, washed once with pre-cooled PBS, dried with absorbent paper, and immediately placed in liquid nitrogen, dry ice, or at −80°C. Samples were packed in dry ice and transported to Hangzhou Jingjie Biotechnology Company (PTM Biolabs, Inc.) for whole protein 4D label-free quantitative proteomic determination. Testes of *Ube2j2*^−/−^ and WT mice were subjected to LC-MS/MS analysis following a standard protocol. The sample was grinded and mixed with lysis buffer (8 M urea, 1% Protease Inhibitor Cocktail III (Merck Millipore, 539134) dissolved in aqueous solution containing buffer salts), followed by sonication on ice using a high intensity ultrasonic processor (Scientz). The remaining debris was removed by centrifugation at 12,000 g at 4°C for 10 min. Then, the supernatant was collected and the protein concentration was determined with BCA kit (Beyotime Biotechnology, P0011) according to the manufacturer’s instructions. The sample was slowly added to a final concentration of 20% (m/v) trichloroacetic acid (Sigma-Aldrich, T4885) to precipitate proteins, then mixed by vortexing and incubated for 2 h at 4°C. The precipitate was collected by centrifugation at 4500 x g for 5 min at 4°C. The precipitated protein was washed 3 times with pre-cooled acetone and dried for 1 min. Protein samples were then redissolved in 200 mM TEAB (Triethylammonium bicarbonate buffer, Sigma-Aldrich, T7408) and ultrasonically dispersed. Trypsin was added at 1:50 trypsin-to-protein mass ratio for the first digestion overnight. The sample was reduced with 5 mM dithiothreitol for 60 min at 37°C and alkylated with 11 mM iodoacetamide for 45 min at room temperature in darkness. Finally, the peptides were desalted using a Strata X SPE column (Phenomenex). The tryptic peptides were fractionated into fractions by high pH reverse-phase HPLC using Agilent 300Extend C18 column (5 μm particles, 4.6 mm ID, 250 mm length). The released peptides were subjected to NSI source followed by tandem mass spectrometry (MS/MS) in Q ExactiveTM Plus (Thermo) coupled online to the UPLC. Peptides were then selected for MS/MS using NCE setting as 28 and the fragments were detected in the Orbitrap at a resolution of 17,500. Maxquant 1.6.15.0 software was used for spectra analysis, database searches, and peptide identification.

### Quantification and statistical analysis

#### Bioinformatics analysis

To analyze differential protein expression, firstly the samples to be compared were selected, the quantitative mean value of modification sites/proteins of repeated samples was calculated, and finally, the fold change of comparison group was calculated. To determine the significance of differences, the relative quantitative values of each modification site/protein in the two comparison pairs were tested by *t*-test, and *P* ≤ 0.05 was used as the default significance index. To make the test data conform to the normal distribution required by the *t*-test, the relative quantitative values of modification sites/proteins were necessary Log2 logarithmic conversion before testing. According to the above difference analysis, when *P* ≤ 0.05, more than 1.5 variation of differential expression was taken as the threshold of significant up-regulation, and less than 1/1.5 as the threshold of significant down-regulation. To obtain a protein uniquely expressed in WT (*Ube2j2*^+/+^ mice) or KO (*Ube2j2*^−/−^ mice), proteins of all WT and all KO groups were summed to obtain proteins uniquely expressed in the WT or KO groups, and a Venn diagram was drawn.

GO (Gene Ontology) and KEGG (Kyoto Encyclopedia of Genes and Genomes) analysis were performed by clusterProfiler (4.0.5) and Metascape (https://metascape.org). Fisher tested and retained significant enrichment GO entries with *P* < 0.01 and Min Overlap = 3. KEGG (https://www.kegg.jp/) integrated molecular signatures, particularly data generated by genome sequencing, to elucidate these functions and interactions. The enrichment of differentially expressed genes in the KEGG pathway was analyzed by Metascape. Enrichment analysis results were plotted using package R clusterProfiler and ggplot2 (3.3.6).

Gene set enrichment analysis (GSEA) was performed using the GSEA (version 4.2.3) method with all protein expression data and phenotypic classification for enrichment analysis to determine whether the pathway’s overall expression form is inhibited or activated. The gene sets with *P* < 0.01 and FDR < 0.1 were considered to be significantly enriched in a pathway.

#### Statistical analysis

Unless stated otherwise, all experiments and statistical analyses in this study were performed with at least three independent biological replicates. Significance tests were conducted using GraphPad Software, and significant differences between groups were determined by two-tailed unpaired Student’s t-tests, with significance defined by ∗*P* < 0.05.

## Supplemental information

Document S1. Figures S1–S4

Table S1. GO & KEGG analysis of differentially down-regulated proteins in the KO group, related to Figure 5

Table S2. Detailed list of unique expression proteins in the WT group for GO enrichment analysis, related to Figure 5

Table S3. Detailed list of unique expression proteins in the KO group for GO enrichment analysis, related to Figure 5

Table S4. Detailed list of genes associated with separation of homologous chromosomes from GSEA, related to Figure 5

Table S5. All protein information identified by Hangzhou Jingjie Biotechnology Company (PTM Biolabs, Inc.) for quantitative proteomic determination, related to Figure 5
