# Supplementary material for: UBE2J2 is essential for the progression of meiosis prophase I during spermatogenesis in mice
Source: iScience. 2025 Jun 11;28(8):112878. doi: 10.1016/j.isci.2025.112878 (PMC12275893; doi:10.1016/j.isci.2025.112878)
Supplement: Document S1. Figures S1–S4 [file mmc1.pdf]

iScience, Volume *28*

Supplemental information

UBE2J2 is essential for the progression of meiosis prophase I during spermatogenesis in mice

Xiaochen Yu, Jie Cen, Yaxuan Zhang, Tongtong Li, Mingyu Zhang, Fei Gao, Hongbin Liu, and Yongzhi Cao

Figure S1

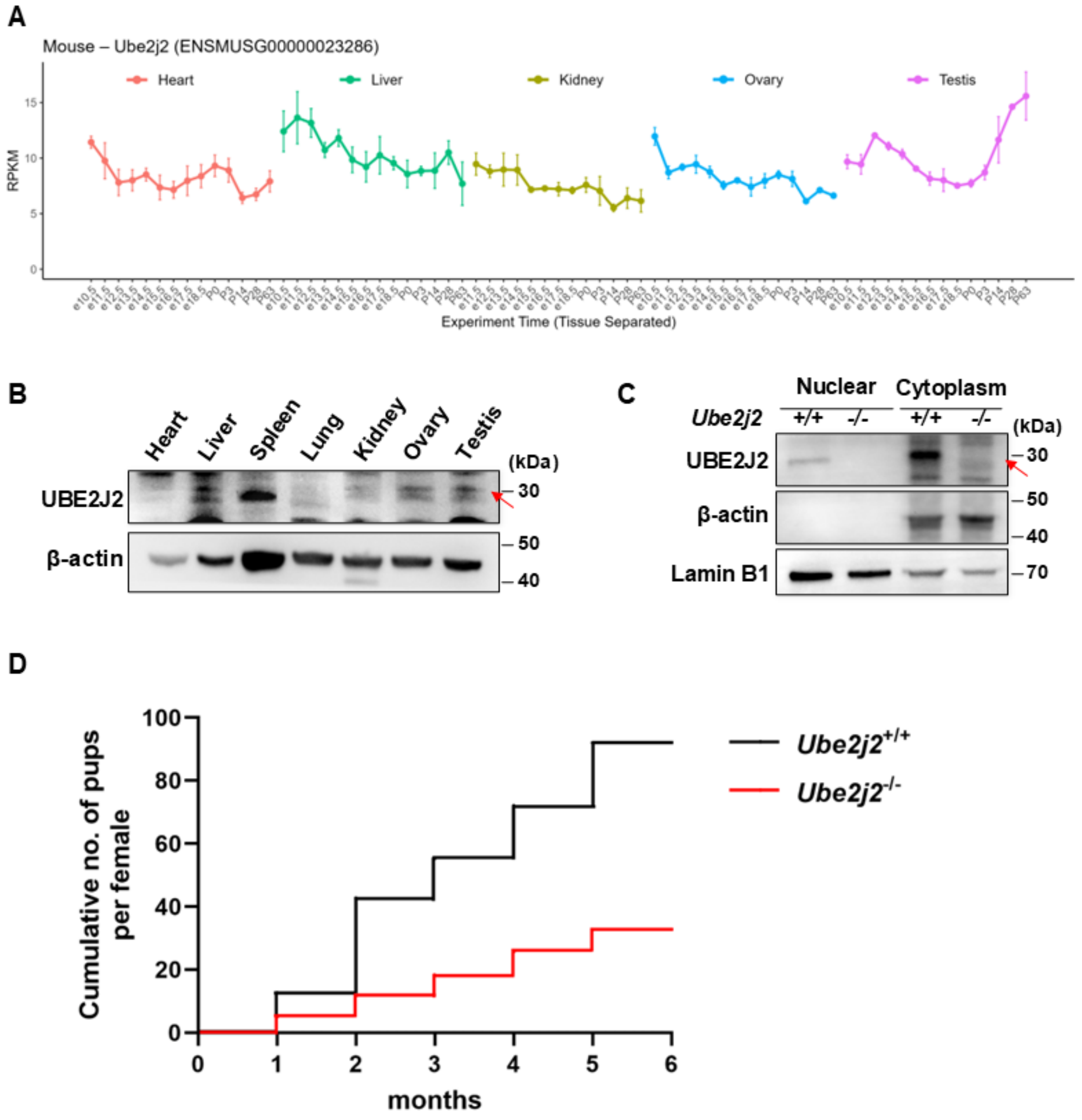

Figure S1. The expression of UBE2J2 protein, related to Figure 1

(A) UBE2J2 expression began to increase significantly in the testis of mice at three days after birth. **(B)** UBE2J2 protein levels in the indicated organs from wild-type mice. β-actin was used as the loading control. Red arrows indicate the positions of molecular marker bands corresponding to UBE2J2. **(C)** Immunoblotting of cytoplasmic and nuclear fractions of PD13 testes from *Ube2j2*$^{+/+}$ mice and *Ube2j2*$^{-/-}$ mice, showing that UBE2J2 is localized in the nucleus and the cytoplasm. Lamin B1 was used as the marker protein for the nuclear fraction, and β-actin was used as the marker for the cytoplasmic fraction. Red arrows indicate the positions of the molecular marker bands corresponding to UBE2J2. **(D)** Fertility tests of *Ube2j2*$^{+/+}$ (n=3) and *Ube2j2*$^{-/-}$ (n=3) female mice.

Figure S2

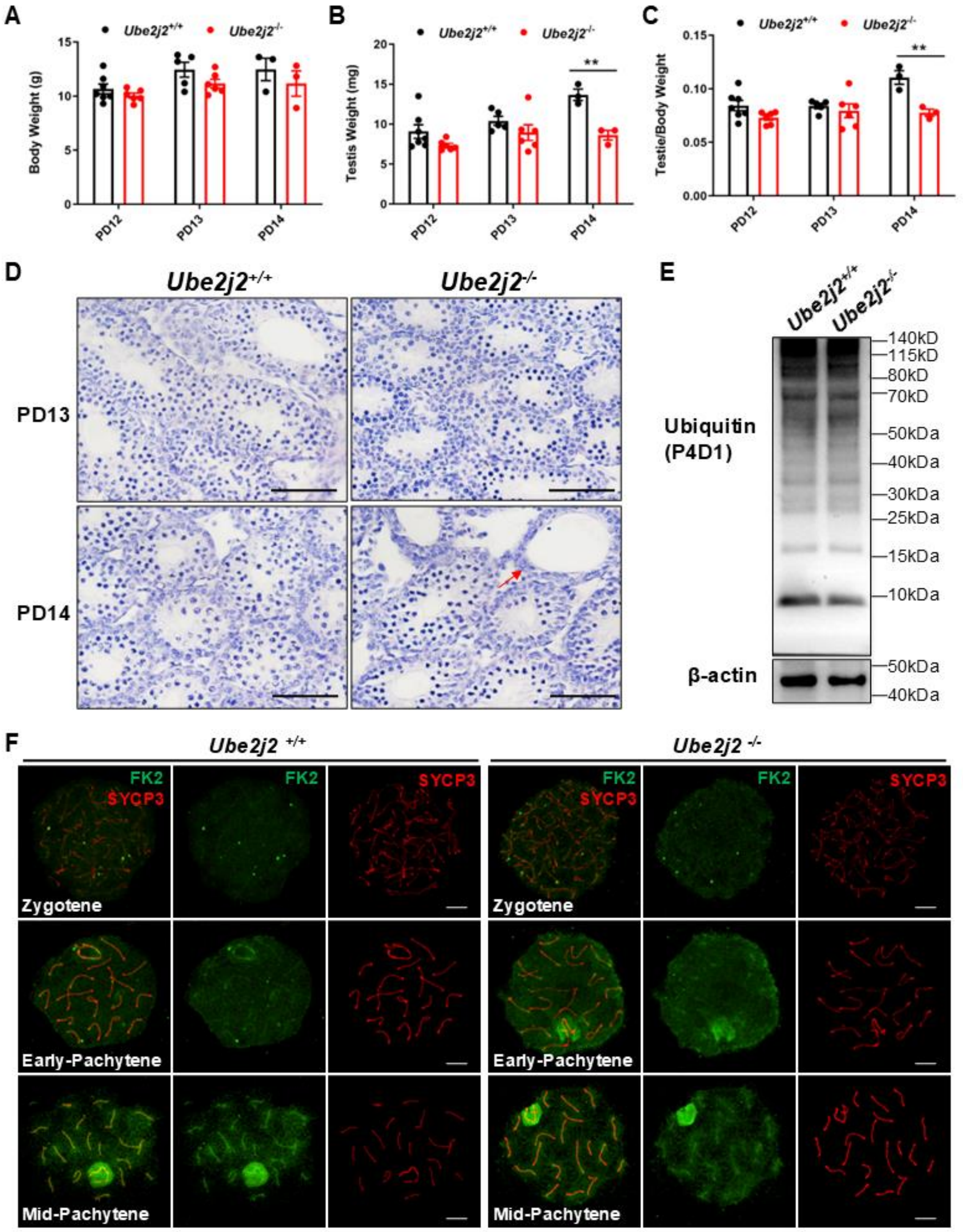

Figure S2. The overall ubiquitination levels are unchanged in *Ube2j2*$^{-/-}$ testes and spermatocytes, related to Figure 5

(A) Body weights of *Ube2j2*$^{+/+}$ and *Ube2j2*$^{-/-}$ mice from PD12-PD14. Columns display means ± SEM. Each dot represents an individual mouse. PD12: n=7, PD13: n=5, PD14: n=3 for both *Ube2j2*$^{+/+}$ and *Ube2j2*$^{-/-}$ genotypes. **(B)** Testes weights of *Ube2j2*$^{+/+}$ and *Ube2j2*$^{-/-}$ mice from PD12-PD14. Columns display means ± SEM. Each dot represents an individual mouse. PD12: n=7, PD13: n=5, PD14: n=3 for both *Ube2j2*$^{+/+}$ and *Ube2j2*$^{-/-}$ genotypes. **, $P < 0.01$, Student's *t*-test. **(C)** Testes/Body weight of *Ube2j2*$^{+/+}$ and *Ube2j2*$^{-/-}$ mice from PD12-PD14. Columns

display means ± SEM. Each dot represents an individual mouse. PD12: n=7, PD13: n=5, PD14: n=3 for both *Ube2j2*$^{+/+}$ and *Ube2j2*$^{-/-}$ genotypes. **, P < 0.01, Student's *t*-test. **(D)** Hematoxylin staining to observe spermatocyte development in testes sections of PD13 and PD14 *Ube2j2*$^{-/-}$ mice. Red arrow indicates a tubule without meiotic spermatocytes. Scale bar, 50 µm. **(E)** Immunoblot detection of non-specific ubiquitin marker, P4D1, in testes samples of PD14 *Ube2j2*$^{+/+}$ and *Ube2j2*$^{-/-}$ mice. Note: P4D1 antibody detects free ubiquitin, ubiquitinated proteins, and polyubiquitin chains. **(F)** Immunofluorescence staining of SYCP3 (red) and ubiquitin chain marker, FK2 (green), in spermatocyte spreads of *Ube2j2*$^{+/+}$ and *Ube2j2*$^{-/-}$ mice. Scale bar, 5 µm.

Figure S3

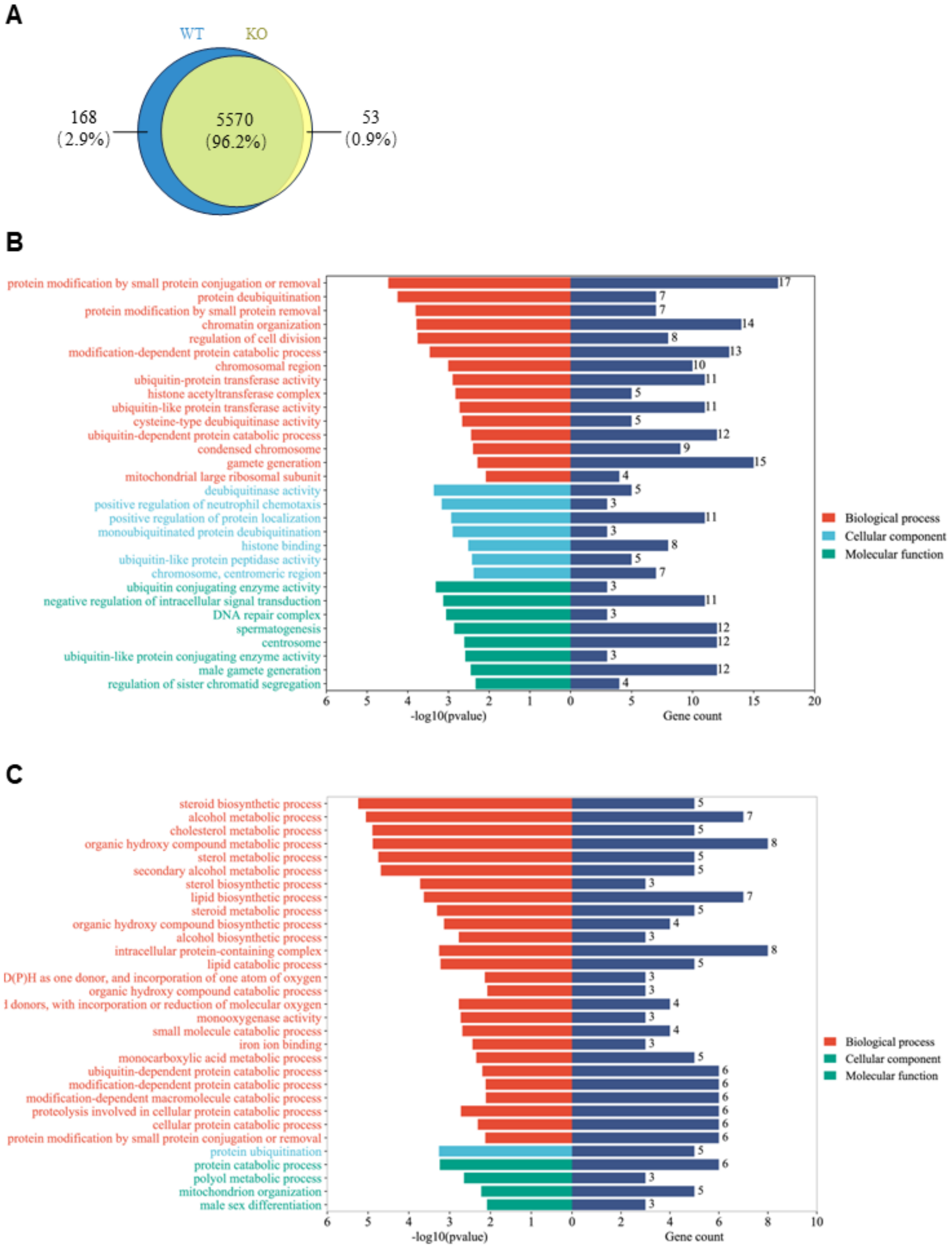

Figure S3. Analysis of proteins expressing only in *Ube2j2*$^{+/+}$ testes and only in *Ube2j2*$^{-/-}$ testes, related to Figure 5

(A) Venn diagram showing overlap of the WT (*Ube2j2*$^{+/+}$ mice) and KO (*Ube2j2*$^{-/-}$ knockout mice) proteins identified in the proteomics analysis of testes, 168 of which were expressed only in the WT group and 53 of which were expressed only in the KO group. **(B)** GO enrichment analysis of unique expression proteins in the WT group for biological process, cellular component and molecular function. **(C)** GO enrichment analysis of unique expression proteins in the KO group for biological process, cellular component, and molecular function.

Figure S4

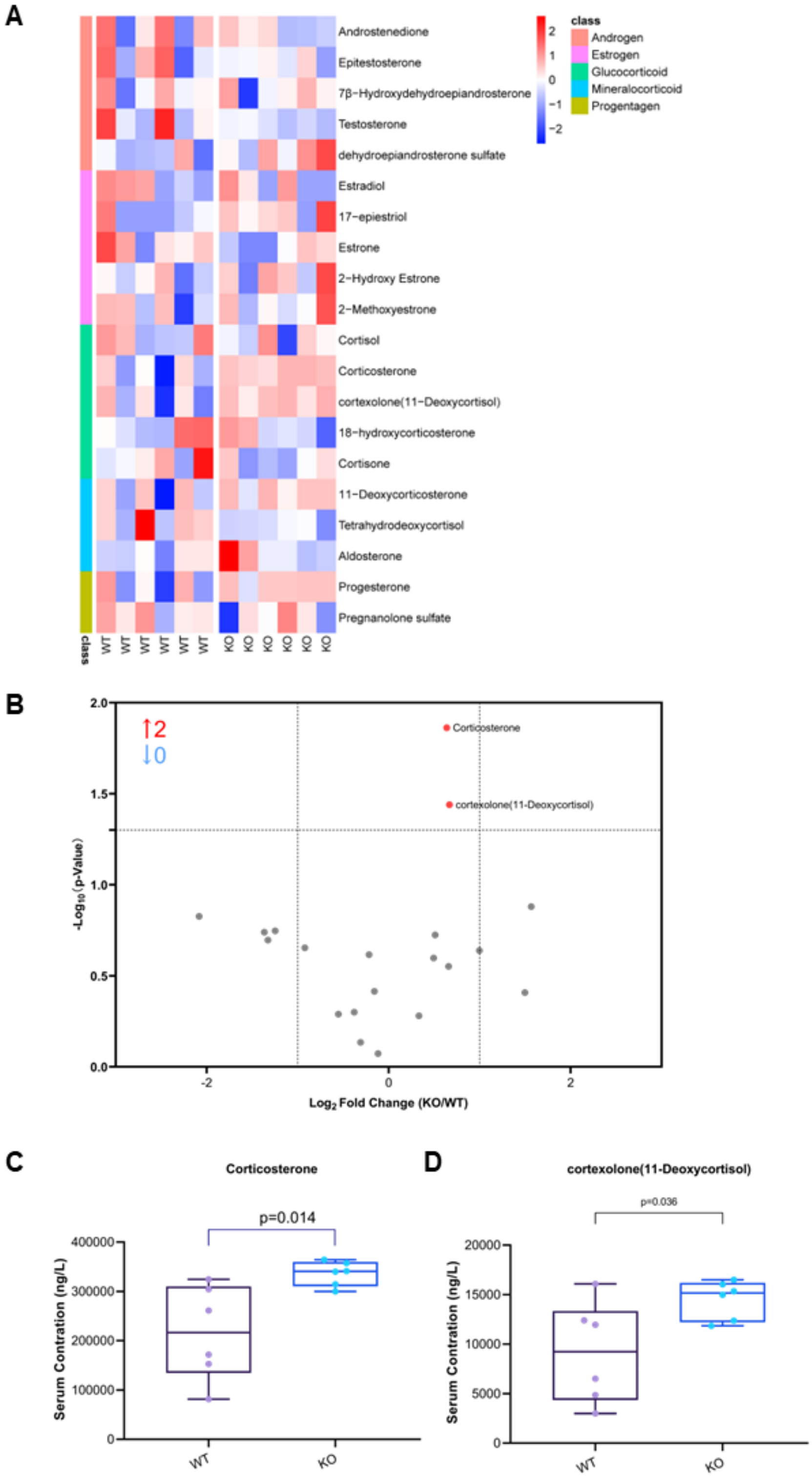

Figure S4. Knockout of *Ube2j2* does not affect the expression of peptide sex hormones, related to Figure 5

(A) The heat map showed the quantitative results of steroid hormones in 6 WT mice and 6 KO mice, including androgen, estrogen, glucocorticoid, mineralocorticoids and progesterone. **(B)** The volcano map showed a significant difference in hormones between the WT and KO groups.

Red represents up-regulated hormone, blue represents down-regulated hormone, and $P < 0.05$. There were two up-regulated hormones and no down-regulated hormones in the KO group. **(C)** The expression of corticosterone in serum samples of KO mice was significantly increased. The statistical method was a *t*-test, with a *P*-value of 0.014. **(D)** The expression of cortexolone in serum samples of KO mice was significantly increased. The statistical method was a *t*-test, with a *P*-value of 0.036.
